# A step-up approach to management of complex bronchopleural fistula

**DOI:** 10.1093/jscr/rjac490

**Published:** 2022-10-31

**Authors:** Kirea J Mazzolini, Jessica M Dzubnar, Jeffrey B Velotta

**Affiliations:** Department of General Surgery, University of San Francisco East Bay, Kaiser Permanente Oakland, Oakland, CA, USA; Department of General Surgery, University of San Francisco East Bay, Kaiser Permanente Oakland, Oakland, CA, USA; Department of Thoracic Surgery, Kaiser Permanente Oakland, Oakland, CA, USA

## Abstract

Bronchopleural fistula (BPF) is a sinus tract between a mainstem, lobar or segmental bronchus and the pleural space. We present a 68-year-old male with a 13 mm spiculated left lower lobe nodule who underwent video-assisted thoracoscopic surgery left lower lobe wedge resection followed by persistent BPF requiring open window thoracostomy. We present a step-up approach to management of persistent BPF with discussion of conservative, operative and reconstructive techniques for closure.

## INTRODUCTION

Bronchopleural fistula (BPF) is a sinus tract between a mainstem, lobar or segmental bronchus and the pleural space [1]. We present a 68-year-old male with a 13 mm spiculated left lower lobe nodule who underwent video-assisted thoracoscopic surgery (VATS) left lower lobe wedge resection followed by persistent BPF requiring open window thoracostomy (OWT). We present a step-up approach to management of persistent BPF with discussion of conservative, operative and reconstructive techniques for closure.

## CASE REPORT

**Figure 1:**
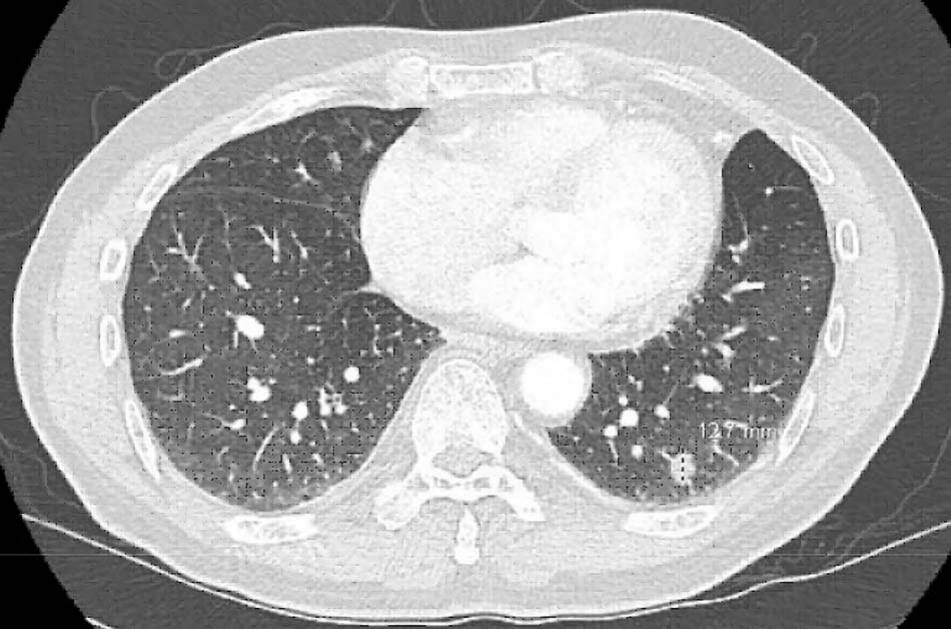
CT scan demonstrating a spiculated left lower lobe nodule.

A 68-year-old male, current smoker with chronic cough and night sweats was diagnosed with a suspicious 13 mm spiculated left lower lobe nodule (Fig. 1). His medical history included a 35+ pack-year smoking history, treated tuberculosis, hypertension, peripheral vascular disease, aortic atherosclerosis and asthma with severe chronic obstructive pulmonary disease (COPD) managed on low-dose steroid taper. Pulmonary function testing and positron-emission tomography (PET) demonstrated a forced expiratory volume (FEV1) of 40% with mildly PET-avid disease. The patient underwent VATS left lower lobe wedge resection of the left lower lobe nodule with mediastinal lymph node sampling. Extensive anthracosis and adhesive disease was seen. Pathology revealed benign parenchyma with fibrinous changes and organizing pneumonia.

**Figure 2:**
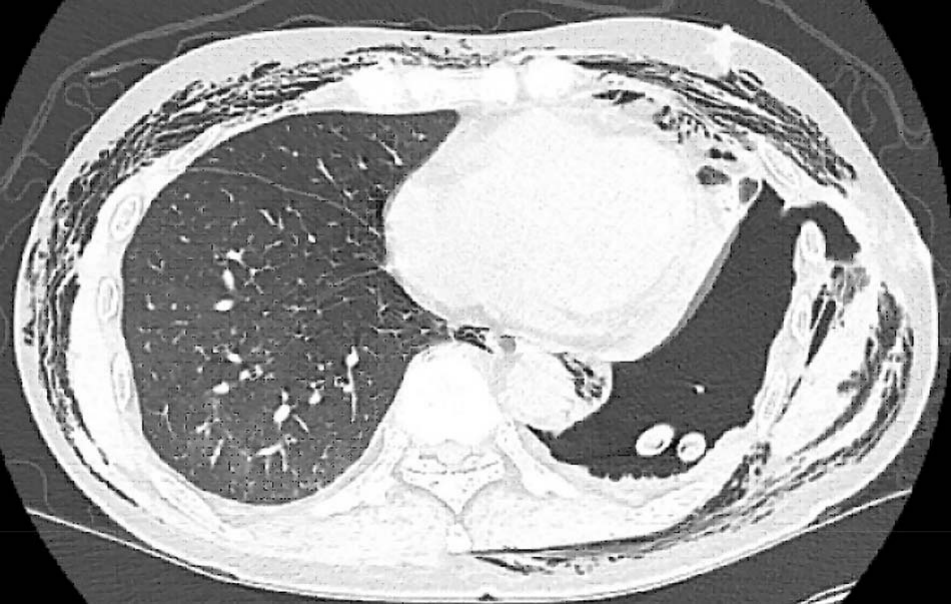
POD13 CT scan demonstrating a BPF between the fifth and sixth ribs communicating with a pneumothorax and subcutaneous emphysema.

Postoperatively, the patient developed persistent expiratory air leak and thus underwent left VATS mechanical and chemical pleurodesis with Doxycycline on POD 4. Air leaks observed at previous staple lines were reinforced with additional staple loads, Floseal and Progel. In addition, an apical pleural tent was performed in the left upper lobe. He continued to have an expiratory air leak. Imaging on POD 13 demonstrated a BPF communicating with the pleural space (Fig. 2). Bedside pleurodesis with Doxycycline was performed through the thoracostomy tube.

**Figure 3:**
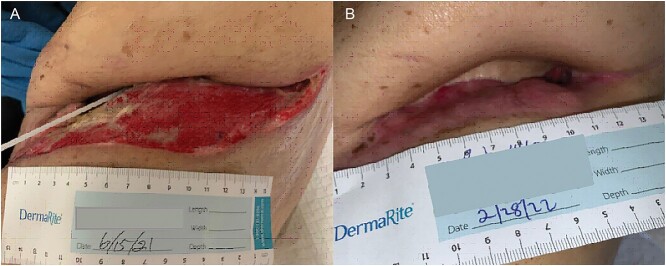
(**A**) POD 19 and (**B**) 9 months status post OWT.

The patient underwent multiple procedures to mitigate the air leak from his peripheral BPF including image-guided 12 Fr. pigtail placement, endobronchial valve placements down the left upper and lower lobe segmental bronchi, and autologous blood patching through the thoracostomy tube. He was treated for associated Aspergillus pneumonia and eventually discharged with stable hydropneumothorax. The patient was readmitted multiple times for chest-wall infection and pneumonia. He underwent OWT via Clagett procedure 5 months after his index operation. Chronic fibrosis and purulent effusion were noted with an air leak at the left upper lobe apex. He was discharged home on POD 23 with wet-to-dry dressings and with an 8-week course of antibiotic and antifungal medication (Fig. 3).

**Figure 4:**
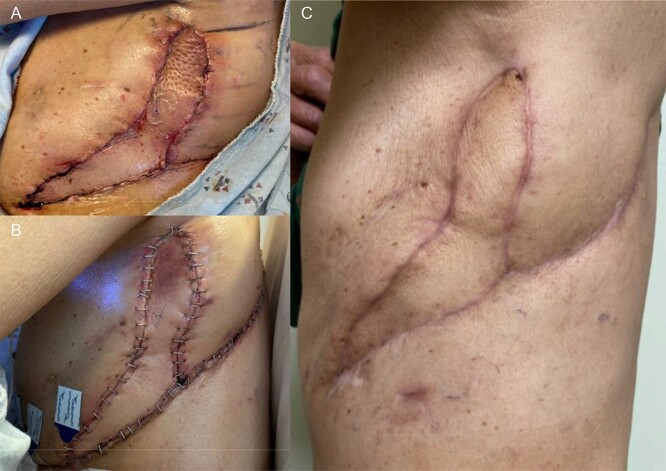
(**A**) POD 0 status post VRAM flap. (**B**) POD1 status post take back. (**C**) About 3 months post VRAM flap.

Once the pleural space infection was eradicated, he underwent definitive BPF closure with a left vertical rectus abdominis myocutaneous (VRAM) free flap alongside Plastic Surgery, 11 months after his first operation. The muscular portion of the flap was secured over the BPF with Vicryl suture, and the skin paddle was used to close the chest-wall. The night of POD 0, he developed subcutaneous emphysema, swelling and flap compromise. He underwent exploration with hematoma evacuation from the subcutaneous space, improving flap perfusion. Two 24 Fr. Blake drains were placed, and the patient was discharged uneventfully. The drains were later removed, and the patient is doing well without respiratory, infectious or BPF issues (Fig. 4).

## DISCUSSION

BPF is an uncommon but highly morbid complication after pulmonary resection. Incidence increases in patients with history of smoking, COPD, poor nutrition, older age, corticosteroid use, infection, malignancy and exposure to chemoradiation [1–3]. Presentation of BPFs is categorized as acute (0–7 days), subacute (8–30 days) and chronic (>30 days). Large, acute BPFs usually result from bronchial dehiscence and require prompt operative intervention. Subacute and chronic BPFs are insidious and often present with infectious symptoms.

As nearly 80% of patients with BPF have associated empyema, broad-spectrum antibiotics and drainage via thoracostomy tube are critical in early management. If these measures do not result in BFP closure, thoracostomy tubes may be used for chemical pleurodesis. Other non-invasive options include blood patching, with an 80–90% success rate, and endobronchial valve placement via bronchoscopy [2, 4, 5]. When conservative measures fail, minimally invasive surgical techniques such as mechanical pleurodesis and pleural tenting can be utilized. OWT is the most invasive but most definitive means of evacuating infection and reducing dead space in cases of significant pulmonary resection [6–8].

This patient underwent each of these interventions after developing an acute parenchymal BPF. His risk factors for the development of BPF include age, tuberculosis, low preoperative FEV1, history of tobacco use with COPD, current steroid use and active organizing pneumonia at the time of his index operation. Though this is an example of an acute parenchymal BPF, its diffuse nature in the setting of underlying lung disease likely contributed to failure of initial minimally invasive operative interventions, leaving OWT as the definitive management option.

After infection clearance, reconstruction after OWT can be performed using skin grafts, local advancement flaps, pedicled myocutaneous flaps or free flaps [6, 9]. Closure of any remaining BPF primarily or with tissue flaps is key to reconstruction. Complications after reconstruction fall into major categories of infection, persistent air leak, bleeding, and impaired respiratory and shoulder mechanics. This patient underwent BPF and chest-wall closure with a VRAM flap with placement of a 19-French Blake drain into his left chest and a 15-French Jackson-Pratt drain at the flap donor site. He developed progressive subcutaneous emphysema, likely driven by residual BPF, and hematoma formation within the flap leading to vascular compromise. Despite little data specifically studying ideal drain size or number, most authors advocate for use of closed suction drains both at the donor site and under the flap to identify and prevent fluid collections that could compromise flap viability or wound healing. When the chest is entered, it is recommended that thoracostomy tubes be left to manage persistent or new air leak [2].

The successful management of this patient’s BPF using a ‘step up’ approach from least to most invasive intervention provides a useful roadmap for the treatment of complex and persistent air leaks. The VRAM flap is one of many techniques for BPF and soft-tissue coverage after OWT. This patient’s postoperative complications show the importance of liberal use of external drains after OWT closure to prevent flap compromise from hematoma, pneumothorax or subcutaneous emphysema in cases where the BPF is not initially completely sealed.
